# Spinal Irritation

**Published:** 1867-11

**Authors:** C. F. Hart


### SPINAL IRRITATION.

By C. F. Hart, M.D.

Mr. Editor:—Of all the diseases that I have ever met with, this is the most strange; assuming various forms and a variety of symptoms.

*June 10th.* Was called to see a negro girl, aged 20; pulse slightly accelerated; respiration quick and short; skin soft and moist; tongue slightly furred; complained of pain in left side. When directed to take a full inspiration, there would be a catch, and she complained of sharp pain under the left breast. There was some nausea, and slight hacking cough; bowels constipated; abdomen quite tumefied. Supposing it to be a mild attack of pleurisy, I directed to be given hydrarg. chl. mit. grs. viij., quinia sulph. grs. ij., and if it did not act during the day, to give about sunset, mag. sulph. ℥ss.; mustard poultice over the affected region, to remain until the pain subsided, or it blistered.

To my great surprise, when I called this evening, the pain had shifted to the right side, extending up near the base of the heart; medicine not acted; had vomited once or twice during the day. Directed the mag. sulph. to be repeated, and in two hours after, if it did not operate, to use an enæma of warm water, and apply the mustard over the right side.

*11th.* The medicine has acted three times during the night, and she feels some better this morning. Pain in the side not troubling her; ate a little toast and drank part of a cup of tea; pulse normal; skin soft; some nausea—for which I directed potassa citras, grs. xx., in solution, every three hours, in a wineglassful of water.

*12th.* Not so well to-day; had rested badly; vomited several times during the night; pain in the side much worse; no cough; pulse 80; tongue heavily furred; skin moist. I now examined the lungs by oscultation and percussion: could detect no lesions; then the heart, the stomach, liver, spleen, and intestines, not being able to detect any abnormal condition of these organs. I then examined the spine, with the same result. No lesions could be detected in any of the viscera; the pulse 80—near the natural standard—and had it not been for the vomiting, I should have concluded it was a case of malingerer. Such a thing as a satisfactory diagnosis, to me, was impossible. Could it be hysteria? Directed she should take a tablespoonful of aqua cal. in same quantity of sweet milk during the day.

*13th.* No better; vomiting continues; pain in the chest more acute. Directed a cantharides plaster, 8x12 in., and continue cal. aqua as before. *7 o'clock P.M.* Blister drawn well; vomiting checked. Directed quinia sulph. extr. hyos., āā, gr. j., every hour, tinc. assafœ. ʒj. every three hours.

*14th.* Rested well last night; took three doses of quin. and hyos., and two of assafœ.; some nausea this morning; continue quin., hyos., assa., also aqua cal. if necessary; blister nearly dry.

*15th.* No change; blister dry.

*16th.* Worse. Symptoms all returned in a more aggravated form; pulse 70; tongue heavily coated; bowels not moved for two days. Gave C. O. salts ℥ss., to be repeated in four hours if necessary; potassa citras grs. xx., in solution, every three hours; mustard poultice to the stomach; made another examination of thorax, bowels, and spine, but could make no farther discovery.

*17th.* No better; vomiting continues; extremities cold; bowels not moved. Gave enæma, with instructions to bathe the extremities with mustard and warm water; continue former treatment. *8 o'clock P.M.* Bowels moved freely; no change in other symptoms; continue treatment.

*18th.* No better; rested badly through the night; vomiting continues; extremities cold; pulse 60, and feeble. Ordered ammonia carb. grs. ij., in aqua ʒj. lavender spts. comp., ʒss., every hour; hydrarg. chl. mit., grs. iv., and repeat in five hours; bathe the extremities and over the liver and stomach with nitro muriatic acid; reapply the blister to the chest. The vomited matter is like the white of an egg, and very sour.

*19th.* Rested better during the night; vomited but twice since yesterday morning; medicine had operated well before night; continue ammonia and lavender comp. spts. every two hours.

*20th.* No improvement.

*21st.* Same.

*22d and 23d.* The same; no perceptible change.

*24th.* Worse.

*25th.* Worse.

*26th.* Worse; to-day, her appetite has entirely failed, indeed there is rather a loathing for food; everything in the way of nourishment causes vomiting. I now insisted on having consultation. The former family physician was sent for, and from him I learned the following facts:—She was a twin sister, had been very delicate from infancy; when one was taken sick, the other would be in the course of from twelve to twenty-four hours after, and both exactly alike; her sister had four years before died with a similar disease to what she was then suffering with, and for several days after, they thought this one would die. The Dr. thought they had probably been poisoned; he now examined the patient very carefully, as I had done before, the thorax, abdomen, and spine, with the same result, (and he was the finest diagnostician that I have ever met with.) I now told him what had been done for her, and how long she had been sick; he thought all had been done that could be, and continued the same treatment and change with the symptoms.

*27th.* No improvement; a loathing for food. Directed essence of beef, tablespoonful every two or three hours, also egg and brandy; changed the carb. for the valerinated tinc. of ammonia; the other treatment the same.

*28th.* Gradually sinking.

*29th, 30th, and 31st.* The same; told her mistress we could do nothing more, and that she would certainly die in a short time—the Dr. ceasing to visit her from to-day.

*July 1st.* No improvement perceptible.

*2d.* Symptoms slightly modified. I again examined her in the same way as before, and this time found, over the third cervical and third dorsal vertebra, some slight tenderness. I dry cupped her from the head to the sacrum, both sides of the spine.

*3d.* Probably some better; took about ℥vj. blood from over the third dorsal vertebra.

*4th.* Improving; vomiting ceased; free of pain; taken some panada to-day.

*5th.* Not so well; nausea returned. Directed a cantharides plaster to be applied to the spine, from the head down to the lower margin of the scapula.

*6th.* Blister drawn badly; rested better than usual; is probably better.

*7th.* Doing tolerably well to-day.

*8th.* Blister dried; does not feel so well.

*9th.* Worse.

*10th.* Pains returned, with vomiting; reapplied blister plaster, and continue ammo. valer. tinc.

*11th.* Better; neither pain or vomiting; blister drawn well; sprinkled with quin. sulph. grs. v., to keep it running; put her on tonic, ferri chl. tinc. and quassia infusion.

*12th.* Improving; continue tonic.

*13th, 14th, and 15th.* Improving, and continued to do so until recovery was complete.

*1863—February 9th.* She was again attacked in the same way, but having the key to her disease, it was not so unmanageable; blistered the spine freely, and continued the same treatment as before, and it yielded in five or six days.

*1864—In June.* She was again attacked, this time very mild, and only lasted her three or four days, and has had excellent health from that time until last August, since which time I have not heard of her. The singularity of the case is its great obscurity, there having been some half dozen or more thorough examinations, and the disease had lasted nearly a month before the least symptom of disease of the spine could be detected.

